# Intergenerational transmission of height in a historical population: From taller mothers to larger offspring at birth (and as adults)

**DOI:** 10.1093/pnasnexus/pgad208

**Published:** 2023-06-19

**Authors:** Joël Floris, Katarina L Matthes, Mathilde Le Vu, Kaspar Staub

**Affiliations:** Institute of Evolutionary Medicine, University of Zurich, CH-8057 Zurich, Switzerland; Institute of History, University of Zurich, CH-8001 Zurich, Switzerland; Institute of Evolutionary Medicine, University of Zurich, CH-8057 Zurich, Switzerland; Institute of Evolutionary Medicine, University of Zurich, CH-8057 Zurich, Switzerland; Institute of Evolutionary Medicine, University of Zurich, CH-8057 Zurich, Switzerland

**Keywords:** stature, birth weight, maternal effects, 19th/20th centuries, trends

## Abstract

Changes in growth and height reflect changes in nutritional status and health. The systematic surveillance of growth can suggest areas for interventions. Moreover, phenotypic variation has a strong intergenerational component. There is a lack of historical family data that can be used to track the transmission of height over subsequent generations. Maternal height is a proxy for conditions experienced by one generation that relates to the health/growth of future generations. Cross-sectional/cohort studies have shown that shorter maternal height is closely associated with lower birth weight of offspring. We analyzed the maternal height and offspring weight at birth in the maternity hospital in Basel, Switzerland, from 1896 to 1939 (*N* = ∼12,000) using generalized additive models (GAMs). We observed that average height of the mothers increased by ∼4 cm across 60 birth years and that average birth weight of their children shows a similarly shaped and upward trend 28 years later. Our final model (adjusted for year, parity, sex of the child, gestational age, and maternal birth year) revealed a significant and almost linear association between maternal height and birth weight. Maternal height was the second most important variable modeling birth weight, after gestational age. In addition, we found a significant association between maternal height and aggregated average height of males from the same birth years at time of conscription, 19 years later. Our results have implications for public health: When (female/maternal) height increases due to improved nutritional status, size at birth—and subsequently also the height in adulthood of the next generation—increases as well. However, the directions of development in this regard may currently differ depending on the world region.

**Significance Statement**

We believe that our historical data exemplify the temporal dynamics of a maternal (height) effect on the offspring phenotype. We add to the existing literature on the intergenerational transmission of wealth and temporal trends in body height by drawing attention to an important aspect of intergenerational dynamics, namely maternal height. When (maternal) height increases in one generation due to improved nutritional status, the height of subsequent generations increases too. In the context of current developments, the trend toward smaller children at birth, which has been observed in many rich countries over the past few decades, could have an impact on current and future trends in adult size. However, the development in this regard may differ depending on the world region.

## Introduction

Since the 19th century, average body height has steadily increased in many parts of the world (1–4). We bring together different lines of literature and exemplify that the trend toward taller people is also the result of an intergenerational maternal height effect on the size of neonates at birth (1, 5). As mothers have grown taller since the 19th century, they have given birth to larger children, who in turn have grown taller as adults. Average height is an indicator of wealth and health status (6, 7). We present additional data that the effects of rising living standards in the parental generation are passed on from the mothers to the next generation and can be observed already at birth (8). Thus, the nutritional status and height of mothers are important for the transmission of wealth and living standards from one generation to the next (9). This finding also has implications for public health initiatives and economic development policy interventions (10, 11).

Factors that are associated with growth and adult height include both genetics and the environment (e.g. nutrition, disease environment, hygiene, physical workload, socioeconomic position [SEP], and hormonal levels) (12–14). The interactions between these factors result in phenotypic variation, which has a strong intergenerational component (i.e. the height phenotype is transmitted from one generation to the next) (5, 14, 15). Human body height is among the first human traits for which the concept of heritability has been analyzed (15–19). Twin studies in affluent populations have estimated the genetic component of variation in human height to be ∼80% (14). Heritability values are, however, variable (15, 20): Family research measuring similarity in height between siblings (rather than twins) and between parents and their offspring has shown that family patterns in growth exist (14, 21). Even in lower-income and historical settings where environmental factors become more important (60–70%), resulting in lower coefficients of heritability, these patterns persist (15, 22, 23).

The intergenerational effects of the parental phenotype are fundamental for various stages of growth (e.g. size at birth, rate of growth, and adult height) (24). According to Perkins et al. (6), maternal height is an exemplary intergenerational factor, since it serves as a proxy for conditions experienced by one generation that affect the health and growth of the next generation. It has been known since the first half of the 20th century that maternal height is associated with offspring size (25–27). Evidence from modern cross-sectional or cohort studies has shown that maternal height may be an important cofactor of offspring health (28), as emphasized by the fact that shorter maternal height is closely associated with smaller size from birth (6) until adulthood (29–33). Thus, when maternal height increases in one generation due to improved nutritional status, the size at birth and adult height of the next generation increase as well (15, 32).

Height trends are most evident in intergenerational comparisons, with parents passing their improved health and height on to their children (15, 34–36). So far, historical data from maternity hospitals have mainly been used to analyze birth weight trends (37–41). Since few maternity hospitals have sufficiently large quantities of continuous maternal height data (42, 43) and the cross-sectional/cohort study designs used in maternal height and offspring size correlation studies, evidence of intergenerational effects of maternal height on offspring size is still limited. Thus, our study aims to enhance our understanding of intergenerational dynamics in height trends over time, using mother–child pairs of the city of Basel, Switzerland, in the years 1896–1939.

## Results

We analyzed maternal height and offspring size of *N* = 12,107 mother–child pairs transcribed from birth records from the maternity hospital in the city of Basel, Switzerland, for the years 1896–1939 (43, 44). Summary statistics of the studied population are displayed in Table S1. Further information on the data and methods can be found in the corresponding section and the Supplementary material. We used generalized additive models (GAMs) in order to allow for potential nonlinear associations (45). Due to a lack of information about SEP for 1927–1931, we performed the analyses without SEP.

**Fig. 1.**
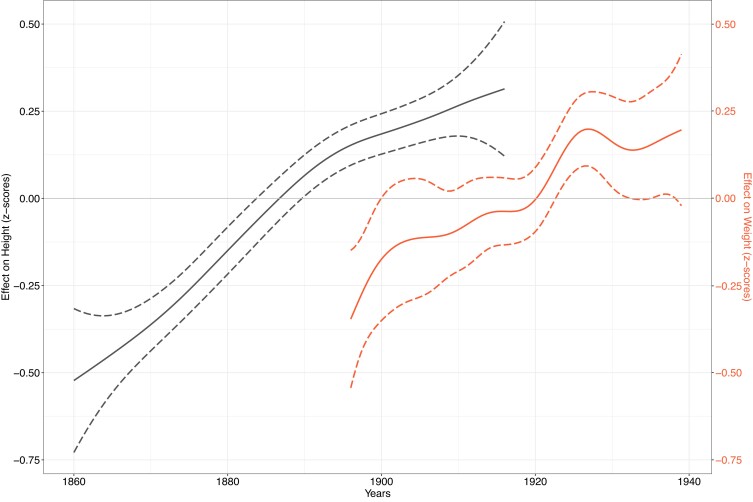
Smoothed association between time (birth years, x-axis), on the one hand, and *z*-transformed maternal height (black line on the left, GAM model 1), as well as *z*-transformed offspring birth weight (red line on the right, GAM model 2, adjusted for all cofactors), on the other (dashed lines = 95% confidence intervals).

**Fig. 2.**
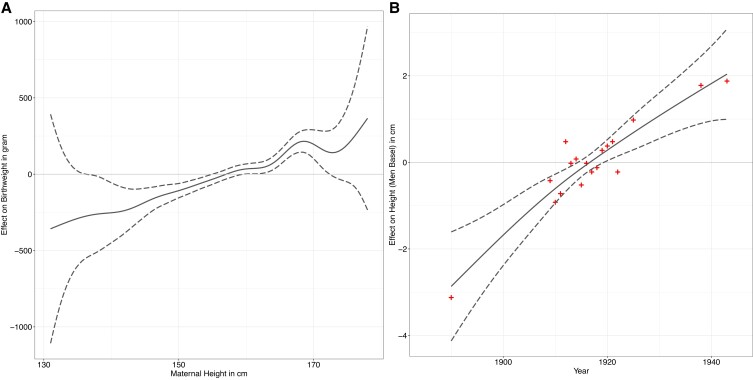
A) The smoothed maternal height effect on birth weight (GAM model 3, adjusted for all cofactors) is almost linear and of considerable size (730 g across the full spectrum of maternal height). B) We find a similarly shaped ∼5-cm increase in average height among men from Basel aged 19 years (GAM model 4, data taken from published conscription averages, >95% coverage) (line = fitted values, dashed lines = 95% confidence intervals, crosses = published data).

The first model estimated the impact of maternal birth year on maternal height, which was *z*-transformed for purposes of comparison and shows a significant (*P* < 0.0001) increase of ∼4 cm in the average height of the mothers born between 1860 and ∼1920 (Fig. 1, black line). The second model estimated the effects of neonatal year of birth, gestational age, parity, neonatal sex, maternal year of birth, and maternal height on birth weight. The smoothed temporal effect of neonatal birth year 1896–1939 on birth weight (once again *z*-transformed for comparison) is also displayed in Fig. 1 (red line) and shows a similarly shaped and significant (*P* < 0.0001) upward trend to maternal height ∼28 birth years earlier. Table 1 reveals that after ranking the independent variables from the birth weight GAM model according to their contribution to the Akaike information criterion (AIC, more information in supplement), maternal height was the second most important explanatory variable after gestational age. A third model estimated the smoothed maternal height effect on birth weight (adjusted for all cofactors), revealing it to be almost linear and of considerable magnitude (up to 200 g per 10 cm increase in maternal height) (Fig. 2A). In the fourth model, we estimated the effect of maternal height on adult height among male conscripts at age 19 and by birth year. Even though this is only approximative, we once again find a significant (*P* < 0.00001) effect on increasing adult male height (Fig. 2B).

**Table 1.** Rating of explanatory factors from the birth weight GAM (model 2) sorted according to their contribution to AIC.

Parameter	ΔAIC
Gestational age	1,816.39
Maternal height	406.48
Parity	298.99
Sex	262.74
Birth year	69.46
Maternal year of birth	16.98

The larger ΔAIC is, the more important the variable is in the model. Maternal height was the second most important explanatory variable after gestational age.

In addition, we performed a sensitivity analysis that took SEP into account for models 1–4, but this resulted in small case numbers for the years 1927–1931. It became clear that the results do not change significantly compared with the results without SEP: Figures show similar patterns whether it is included or not (Figs. S4 and S5, compared respectively to Figs. 1 and 2), and the sorting of explanatory factors according to contribution to AIC ranks SEP variable as the least important, meaning it does not add any additional value to the model 2 explaining birth weight (Table S2). Three additional sensitivity analyses were performed: First, the data were restricted to first-parity births to exclude potential multiple entries for individual mothers, which produced a similar result to Fig. 1 (Fig. S6). Second, the analysis was stratified into SEP groups (low, middle, high, and housewife) to address a potential sample selection bias prior to World War I, when <50% of all births in Basel were covered by the maternity hospital data. Once again, the results (Fig. S7) are similar to Fig. 1. Third, we stratified the adjusted models for the smoothed time trend (Fig. S8) and the smoothed maternal height effect (Fig. S9) by sex, but no sex differences were found.

## Discussion

Our results show that average height of mothers in Basel increased by ∼4 cm across 60 birth years, as did the average birth weight of their children 28 years later. We also found a significant and almost linear association between maternal height and birth weight. Maternal height was the second most important variable in modeling birth weight, following gestational age. Additionally, a significant association between maternal height and the aggregated average height of males from the same birth years was observed when they were conscripted 19 years later.

The increase in the average height of mothers born from the 1860s onward, as well as of 19-year-old conscripts in Basel born after the 1890s, has already been shown and discussed in previous studies (46, 47). Here, we suggest that these trends are probably intergenerationally linked and thus potentially also causally linked: The precondition for the increase in adult height among males born after the 1890s may have been an increase in the height of their mothers who were born after the 1860s. We argue that the increase in average adult height of these males can also be attributed to maternal height and living standards, indicated by larger offspring size at birth. Due to limited availability of historical data containing information on maternal height, result comparison with other data sets and literature is constrained. When maternal height was present in smaller historical samples (e.g. from New York), it had a strong influence on birth weight (48). Evidence from 19th and early 20th century intergenerational family data has shown that there were strong height correlations between brothers, or effects of sibship size (49–52), thus emphasizing the importance of the familial perspective. From a broader point of view, our results are in line with other historical studies that have found strong intergenerational effects of general health, social mobility, and poverty in 19th century populations (53–55). Our results also find correspondence in studies on primates. For example, matrilineal transmission of birth weight has also been described in the rhesus monkey across several generations (56). Interestingly, birth weights of male offspring increased earlier across generations compared with female offspring. However, the models stratified by sex of offspring in our human data from Basel did not show such a difference.

The fact that maternal height is associated with offspring birth weight and postnatal growth is well known from modern cohort and survey studies in both high- and low-income countries (11, 29, 36, 45, 57–66). Fetal and maternal genetic factors contribute to birth weight (65); however, researchers have only recently started to better understand their complex interplay in shaping the associations between maternal height and offspring birth weight (67–69). Mendelian randomization analyses have shown that the association between maternal height and fetal growth is mostly defined by genetics, through the transmission of height-associated single-nucleotide polymorphisms (70). Still, other Mendelian randomization studies have shown that this causal association persists independently of the height alleles transmitted: Fetal growth is first and foremost impacted by genetics factors, but other factors are also in play (69). Potential causal pathways include greater availability of space for fetal growth, causal associations between height and SEP, and assortative mating (69). Women who are shorter may have reduced protein and energy reservoirs, smaller reproductive organs, and limited room for fetal growth, which can affect fetal growth via the placenta (9, 29). Another similar mechanistic aspect could be that small uterine size is more frequent among short mothers, which can lead to stretching of the membranes, shortening of the cervix, or other biological reasons that can increase the chances of preterm birth and low birth weight (65).

While genetic factors may be more dominant in such well-nourished populations, it is important to be more reserved in the context of historical populations where nutritional status has clearly played a major role in the secular trend (71). This has been well documented in a number of studies from the 1970s to the 1990s. Going back even further, the classic 1930s crossbreeding experiments of Walton and Hammond (72) first brought attention to maternal size and its role in fetal growth—a phenomenon later known as maternal constraint, which is an important physiological cause of the variation in birth size, especially at the lower extremes (73, 74). Human data from embryo transfer experiments also reemphasized the role of recipient maternal weight and height (75). The mechanisms have been discussed and investigated: for example, reduced uterine size leading to small growth in the next generation (76), fetal–maternal conflict (77), and other processes such as those related to epigenetics.

The discussion of the influence of nongenetic factors goes back to the intergenerational hypothesis proposed by Emanuel in 1986, in which conditions experienced by one generation relate to growth and health of the next generation (78, 79). In this context, adaptive maternal effects have been discussed since the 1990s (80) and denote causal effects of the maternal phenotype on its offspring's phenotype (nongenetic inheritance) (81). A possible pathway for maternal effects is adaptive phenotypic plasticity across generations, where the environmental exposure that generated the mother's phenotype is translated directly into the phenotype of the offspring (81, 82). The covariance of parental and offspring phenotype is driven by a variety of possible mechanisms (15, 24): Developmental plasticity adjusts the phenotype of the offspring to the actual/predicted environment and to the maternal capital/phenotype (24). The “developmental origins” hypothesis emphasizes the role of epigenetic and other maternal effects in the prenatal and postnatal period as a “fine-tuning” mechanism to adapt to a predicted environment (83–85). In an environment perceived as threatening and unstable, with a predictably uncertain life course, fetal programming (85–88) and epigenetic effects (89) lead to adjustments in order to ensure survival or reproduction (resulting in smaller adult size). Moreover, life history theory focuses on the shaping of an individual's energy/resource allocation strategies throughout their life course and is a promising concept to explain adaptive plasticity and growth/adult size (15, 90, 91).

Height in adulthood reflects a mother's health stock cumulation throughout her childhood and adolescence mostly from her nutritional and socioeconomic statuses and health environment in early childhood (11, 92). Some studies suggest that it is primarily the mother's own birth weight rather than her adult height that is associated with the birth weight of her offspring (35, 93, 94). This would, in turn, point back to the grandmother's nutritional status during pregnancy as an important contributor. Short maternal height is likely to be partially caused by deprivation before and after the mother's own birth and therefore linked to her own mother's nutritional status and standard of living (5). However, there is also some evidence that the postnatal growth experienced by mothers in their childhood is correlated with the birth weight of their offspring, with leg length being the most important component (95). This is in line with studies showing that leg length is a sensitive indicator for childhood nutrition (96, 97) and that the secular increase in adult height mainly reflects a greater increase in leg length as against trunk length (98). To summarize, birth weight is influenced by many maternal phenotypic traits, which, together, form the maternal capital. If maternal stature is a major maternal capital component, creating a constraint on offspring birth size through uterine volume, other parameters such as maternal lean mass can affect birth weight. The accumulation of maternal capital throughout her life course also plays a role: the mother's own birth weight, her stature during childhood or body mass index (BMI) in adulthood influence her offspring's birth weight, independently of maternal adult stature (99, 100).

It is important to emphasize that the heritability of birth weight is highly context specific. High heritability and high genetic influences tend to operate in the context of high-income societies. There is evidence that in other contexts (depending on the world region or time period), the genetic influence may be considerably lower (101, 102). In addition, nutrition-related stunting and other relevant environmental factors may have a greater impact especially in nonhigh-income regions. In general, the confounding effect of better nutrition over two generations cannot be excluded, as birth weight is influenced by maternal nutrition over 9 months, and childhood stunting often occurs in the first 4 years of life. Thus, a 5-year period of nutritional change may affect both birth weight and the likelihood of stunting (which in turn affects adult height).

Our study has several limitations: We only know the average adult height of male offspring at 19 years of age, born in the maternity hospital thanks to published aggregated data from conscription, which also includes the male offspring of mothers who gave birth outside of the maternity hospital. Information on adult height for the female offspring is completely missing. As the historical Swiss conscription data have very high coverage (over 95% of a male birth cohort) (103, 104), the potential for socioeconomic selection bias is relatively low. Regarding the lacking female offspring's adult height, available female height data in Switzerland (46, 105) show that although the expected sexual height dimorphism is there, the trends for the two sexes always go in the same direction, even in the first half of the 20th century. Therefore, there is no reason to assume that the height trend pattern for women later in adulthood should be different from that of men. Likewise, maternal, infant, or child mortality could influence the strength of the associations between birth weight and later adult height, either through selection (in the sense that weaker children died and no longer negatively influence average adult height) or also through scarring (in the sense that weaker children increasingly survive and later negatively affect average adult height) (106). The relevance of these possible effects for the present results must remain unanswered at the moment. However, maternal mortality was a great rarity in the Basel maternity hospital at the beginning of the 20th century. Furthermore, the archival records do not provide any information on paternal height, which would also have an effect on offspring size. Information on maternal weight before and during pregnancy is missing, although it is known that maternal BMI and weight are important components of the maternal capital affecting birth weight (99). This aspect would be relevant, considering that obesity was hardly and undernutrition was more common in the first half of the 20th century in Switzerland, in contrast to the obesity epidemic we find ourselves in today (104). Moreover, prior to World War I, our sample composition might be biased toward the lower SEP strata (i.e. lower population coverage due to higher rates of missed home births, especially among higher SEP parents). However, our sensitivity analysis showed that the same temporal trends occurred at all SEP levels.

Cross-sectional studies have been the main source of documentation of secular trends in average height, mainly for males (66). We add to the literature by tracking intergenerational changes in height and offspring size along the female/maternal line. We also show how health and nutrition of one generation contributes, through maternal transmission as well as through infant and childhood experience, to the health of the next generation, a process lasting several generations (5). We follow Oxley (9) in showing that female height matters as a bridge that transfers welfare and living standards from one generation to the next. Mothers thus play a very important role: Their adult height not only records their accumulated nutritional and health history but also shapes the next generation (including their future economic performance), thus establishing health dynasties. Women should, in reality, occupy an even more central role in economic development.

We believe that our historical data exemplify the temporal dynamics of a maternal (height) effect on the offspring phenotype (107). However, the genetic and nongenetic pathways of this association are not yet fully understood. We add to the existing literature on the intergenerational transmission of wealth (108, 109) and temporal trends in body height by drawing attention to an important and previously neglected aspect of intergenerational dynamics, namely maternal height. Our results may have implications for research on height and health trends in past and present populations: When (maternal) height increases in one generation due to improved nutritional status, the height of subsequent generations increases as well (5). However, the directions of development in this regard may currently differ depending on the world region. In the context of current developments, the trend toward smaller children at birth, which has been observed in many developed countries over the past few decades, could have an impact on current and future trends in adult size (110).

## Materials and methods

### Historical context: Basel ∼1890–1950

As shown elsewhere (43, 111), during the late 19th and early 20th centuries, Switzerland experienced major social and economic transformations associated with industrialization, rapid urbanization and the emergence of the service sector (112). Already by the end of the 19th century, the gross domestic product (GDP) of Switzerland ranked it among the most prosperous countries in Europe (113). Basel City, which consists of the city of Basel and two small surrounding municipalities, experienced rapid growth after 1850. Starting out as a traditional trading town, Basel developed into a leading chemical and pharmaceutical center (47). From 1896 to 1939, the city's population nearly doubled, rising from 89,103 to 162,600 (Fig. S1A).

With the exception of the First World War, conventional measures of living standards (both monetary and health related) show a continuous rise in general wealth in Basel during the period 1895–1940 (43, 114). Skilled workers’ real wages in Basel (Fig. S1B) have doubled (115, 116). The infant mortality rate in Switzerland (Fig. S1C), which was higher in urban than in rural areas, decreased at a steady rate in Basel, as did the stillbirth rate (Fig. S1D) (117). Consequently, life expectancy increased (114, 118). Infectious disease deaths have been falling since the second half of the 19th century, partly due to large improvements in sanitary conditions: Clean water and flush sewage systems have been available since the 1860s and expanded constantly since that time (117). Basel has been undergoing a demographic transition since the last quarter of the 19th century, when the number of deaths and then births per 1,000 inhabitants began to fall (Fig. S1E).

### Data source: Birth records from the maternity hospital

We make use of a historical birth-weight database, which was transcribed and developed in the course of a completed Swiss National Science Foundation project (No. 156683) (43, 44, 46, 111). As the present source and data have already been described, we provide here only a brief summary. The individual data for this research were obtained from the birth records of the University Maternity Hospital of the Canton of Basel-Stadt (Staatsarchiv BS, Sanität X29). For every birth, detailed information has been routinely registered from 1896 onwards. Each birth record in the whole series of register books provides comprehensive and detailed description of the mother, the newborn child, and the delivery. However, the books available for the period after 1900—between one and three per year—only represent a third of those that were initially produced (due to lack of storage space, the archives had to reduce the collection of books in the 1970s) (44). The register books are carefully preserved, and incomplete records are very rare.

Founded in 1868, the maternity hospital was also a teaching hospital (119). Throughout the observation period, the majority of patients came from Basel. In 1897, ∼30% of all births in Basel took place in the maternity hospital (Fig. S1F). As early as 1912, the number of births at the hospital increased to over 50%, while the proportion rose to over 65% following the war, reaching ∼75% by 1930 (111, 120). The Basel birth registry includes births to women from both the top and bottom of the socioeconomic spectrum, as well as difficult and normal deliveries (111).

On the whole, the time period under consideration witnessed a rising standard of living with increasing real wages and decreasing infant mortality and stillbirth rates (Fig. S1). The advantage of using this data set is that maternal height is recorded in >80% of cases (111). Compared with other hospital data sets from the early 20th century, there is no obvious socioeconomic bias with regard to the mothers who delivered in Basel (111). However, our data set does have a few gaps: Information from births in 1900–1905 is missing, occupational information for the parents is missing for 1927–1931, and occupational information for fathers is missing for 1935–1937. More information regarding available data and sources, along with their limitations, can be found in the Supplementary material.

Nearly all birth records were linked to another register by means of a continuous and unique entry number (inventory Sanität X8). From this second register, additional variables regarding the socioeconomic background of the father (if known) are available. The occupations were classified by SEP as proposed by Schüren (121). We used the SEP of the father to measure the SEP of the family. Where this was not possible, the SEP of the mother was used. A comparison of the breakdown of paternal occupations in the maternity hospital data with the census results from 1910 for male occupation groups shows that they are very similar (111). This suggests that families whose mothers gave birth at the maternity hospital may be a representative sample of the whole Basel population. In the years 1935–1937, many mothers are categorized as housewives. The majority of the fathers have a medium SEP. The share of lower SEP fathers decreases slightly over time while the share of upper SEP fathers increases slightly. Permission to access the protected individual data was granted by the Staatsarchiv Basel-Stadt upon signing a contractual agreement. After linking the sources and data cleaning, the data were fully anonymized.

Despite extensive archival research, we have not been able to find an official measurement protocol from the maternity hospital for anthropometric measurements such as birth weight or maternal height. There is also no reference to these measurement procedures in other reports of the hospital. The exact measurement practice must therefore remain uncertain and could have changed over time as well as between measuring persons. The distribution graphs of birth weight and maternal height (Fig. S2) suggest, by their tendency toward symmetry and lack of strong rounding effects, that these data were measured and not estimated. This is even more certain for birth weight, where scales were obviously used for measurement.

We linked variables about the father (if known) from the birth register to variables about the mothers and their offspring found in the birth records (see Supplementary material). We used the father's occupation as a proxy for his SEP and that of his family. When this was not possible, we used the mother's occupation. The breakdown of paternal occupations in the data set closely matched the values in the male occupation census from 1910 (111). The density plots of maternal height, birth weight, birth length, and gestational age show the expected tendency toward symmetry (Fig. S2). In terms of sample composition, the shares of primiparous women and of parents with a higher or medium SEP increased over time (Fig. S3). This reflects the demographic transition toward a smaller family model, as well as a general increase in living standards.

From the initial sample (*N* = 13,988), we excluded the years 1901 and 1902, since only first parities were transcribed in those 2 years (*N* = 238, 1.7%). We also excluded births for which the maternal year of birth was 1917–1919, because only a few births were recorded for these values (*N* = 734, 5.2%). Multiple births (*N* = 321, 2.3%), stillborn neonates (*N* = 592, 4.4%), mothers aged <20 years (*N* = 311, 2.5%), missing birth weight (*N* = 44, 0.3%), missing parity (*N* = 18, 0.1%), missing maternal age (*N* = 3, <0.1%), missing newborn sex (*N* = 2, <0.1%), and missing gestational age (*N* = 352, 2.8%) were excluded as well, resulting in a sample size of *N* = 12,107 (86.6% of the initial sample). In order to estimate how tall the newborn males had grown by the time they were recruited at age 19, we used published mean values (47, 122), as individual matching was not possible.

### Statistical analyses

To examine the nonlinear associations between maternal height or year of birth and birth weight, we applied general additive models (GAMs) (123, 124). By allowing not only linear associations but also general smooth terms, GAM models are an extension of generalized linear models (GLMs). This allows nonlinear relationships between outcome and exploratory variables to be fitted (in addition, the smooth terms reduce to basically linear associations if the data do not require nonlinearity). We fit a combination of linear and smooth terms in our models. We used smooth terms for the variables: year, maternal year of birth, maternal height, and gestational age. We used linear terms for the variables: sex and parity. All linear terms were tested for multicollinearity by calculation of the variance inflation factor, and the smoothed terms were tested by measurement of concurvity. None of the variance inflation factors were >1.5, and the concurvity indices were <0.15. Therefore, all variables were regarded as noncollinear.

In order to explore the importance of each independent variable on the outcome, each independent variable was dropped from the model one at a time and the AIC of the model was computed. We then computed the difference between the AIC for the complete model M and the model with one independent variable k removed, i.e. ΔAIC_k_ = AIC_k_ − AIC_M_. The larger ΔAIC_k_ is, the greater the importance of the variable in the model.

R version 4.2.1 was used for all statistical analyses. The package “mgcv” (123) was used for the GAMs, and “ggplot2” was used (125) to produce all figures.

## Supplementary Material

pgad208_Supplementary_Data

## Data Availability

The data used in this study are uploaded as a Supplementary material.
