# Supplementary material for: Intergenerational transmission of height in a historical population: From taller mothers to larger offspring at birth (and as adults)
Source: PNAS Nexus. 2023 Jun 19;2(6):pgad208. doi: 10.1093/pnasnexus/pgad208 (PMC10306274; doi:10.1093/pnasnexus/pgad208)
Supplement: pgad208_Supplementary_Data [file pgad208_supplementary_data.pdf]

Supplementary Material:

Intergenerational transmission of height across time: From taller mothers to larger offspring at birth (and as adults)

Figure S1: Selected contextual variables mapping socio-demographic changes in Basel between about 1890 and 1940.

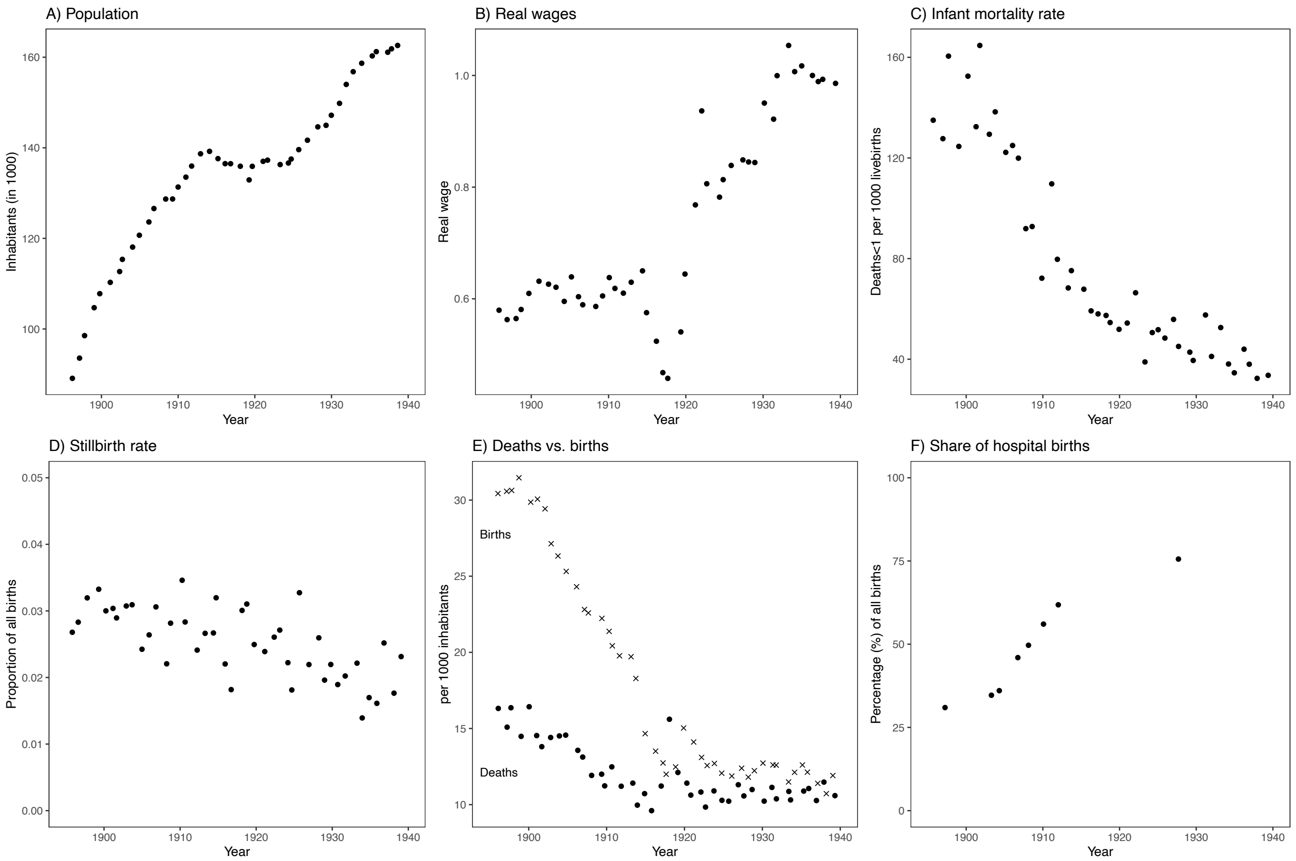

Figure S2: Kernel density plots indicating the smoothed distributions for maternal height, birthweight, birth length, and gestational age.

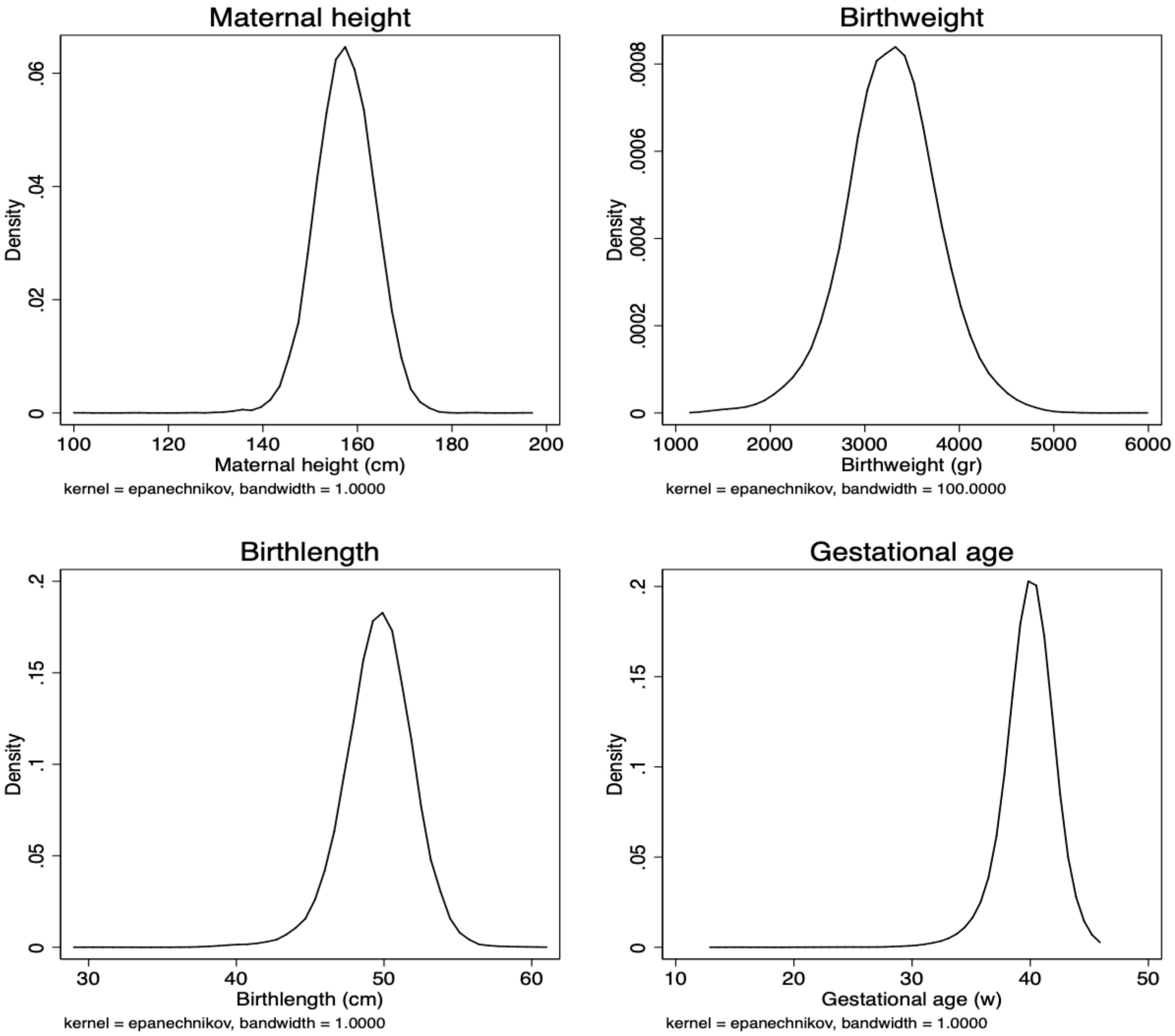

Figure S3: Annual sample composition according to offspring sex (A), SEP (B), and parity (C). Boxplots indicate the annual distributions for maternal age (D), gestational age (E), maternal height (F), and birthweight (G). The years 1900–1905 are missing from the data set.

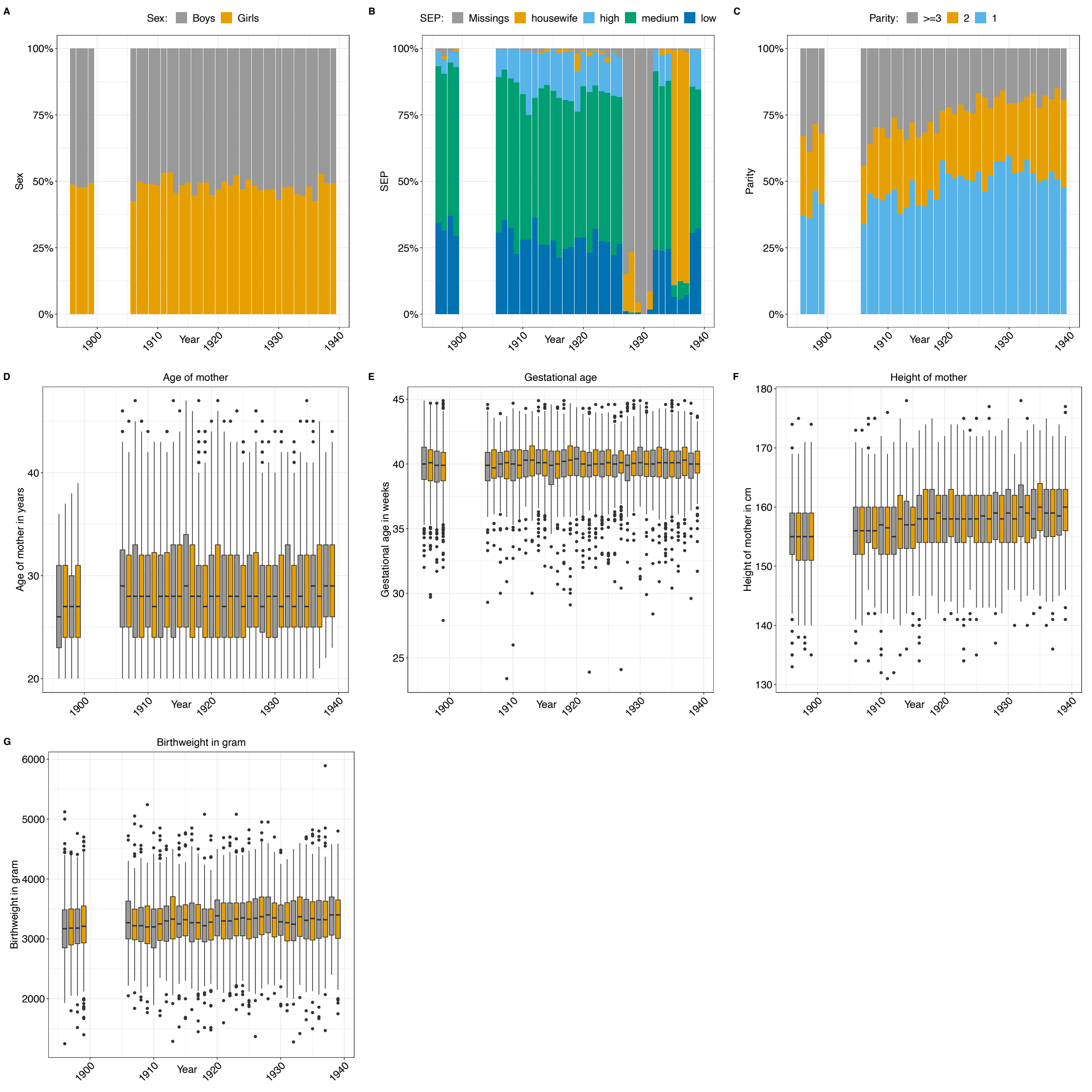

Figure S4: Same models as in Figure 1, but with SEP as a sensitivity analysis.

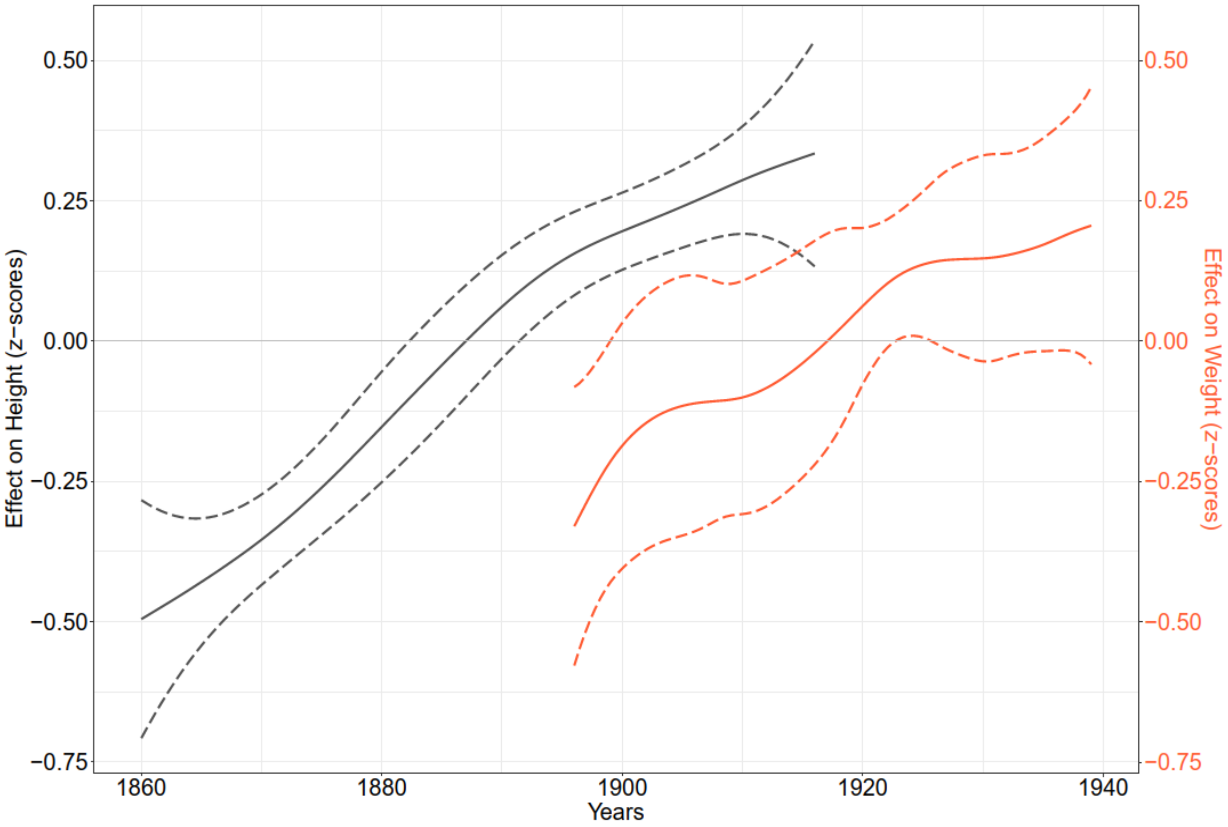

Figure S5: Same models as in Figure 2, but with SEP as a sensitivity analysis.

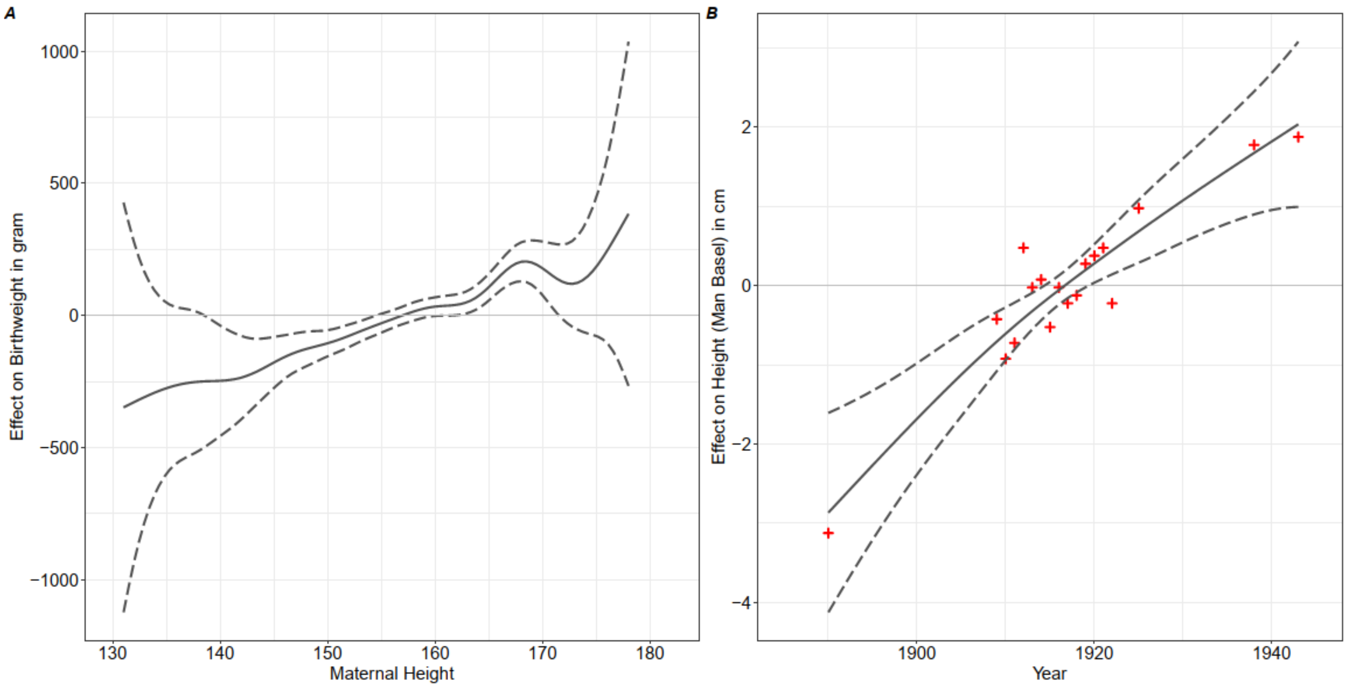

Figure S6: Same models as in Figure 1, but for first-parity births only as a sensitivity analysis to account for the possibility of multiple entries for the same women in the data set.

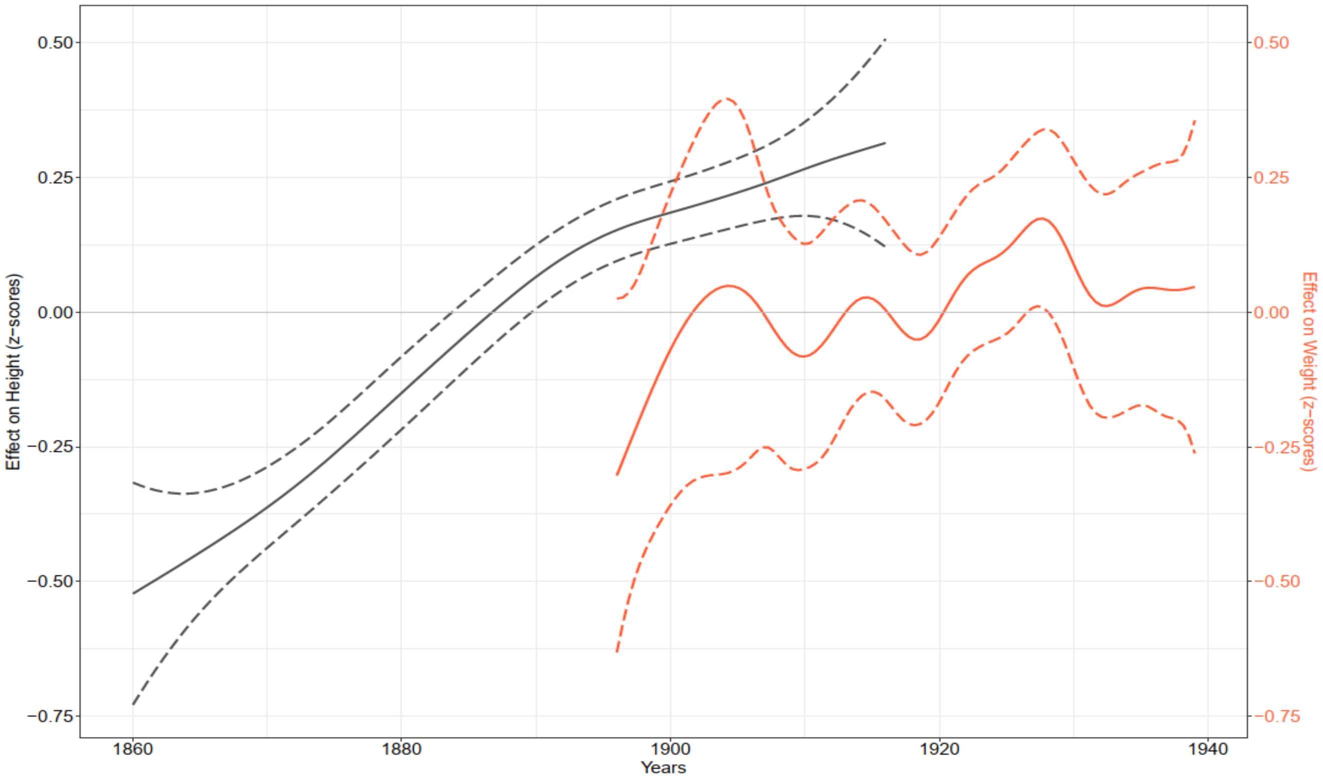

Figure S7: Same models as in Figure 1, but stratified by SEP groups. Please note that the “high” SEP group is small in size, and that the “housewife” group mostly appeared only in the years 1935–37 (when most of the father’s occupations were missing).

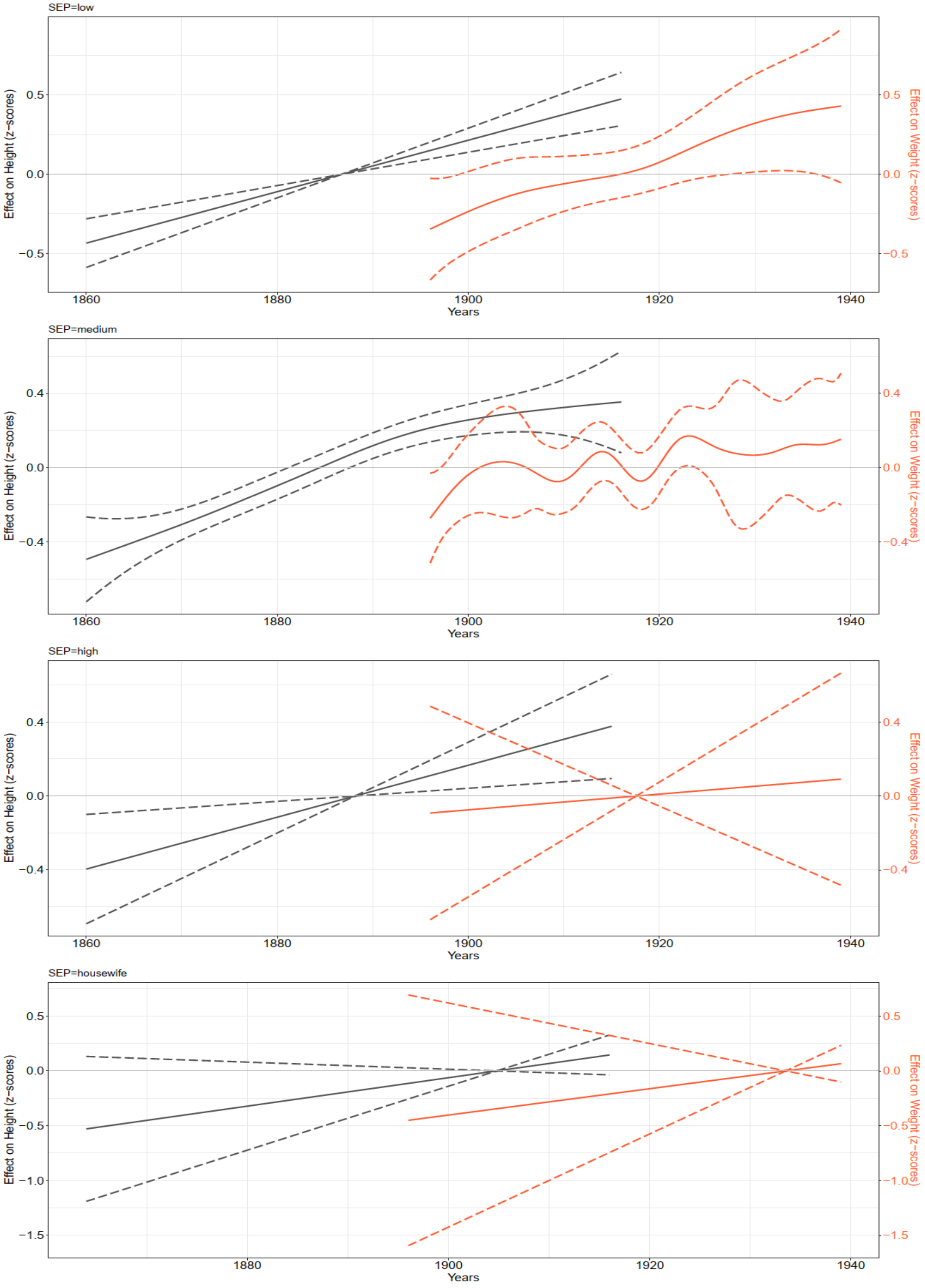

Figure S8: The adjusted model for the temporal trend, stratified by sex. A) The dependent variable birthweight displayed as grams; B) the dependent variable birthweight displayed as z-scores.

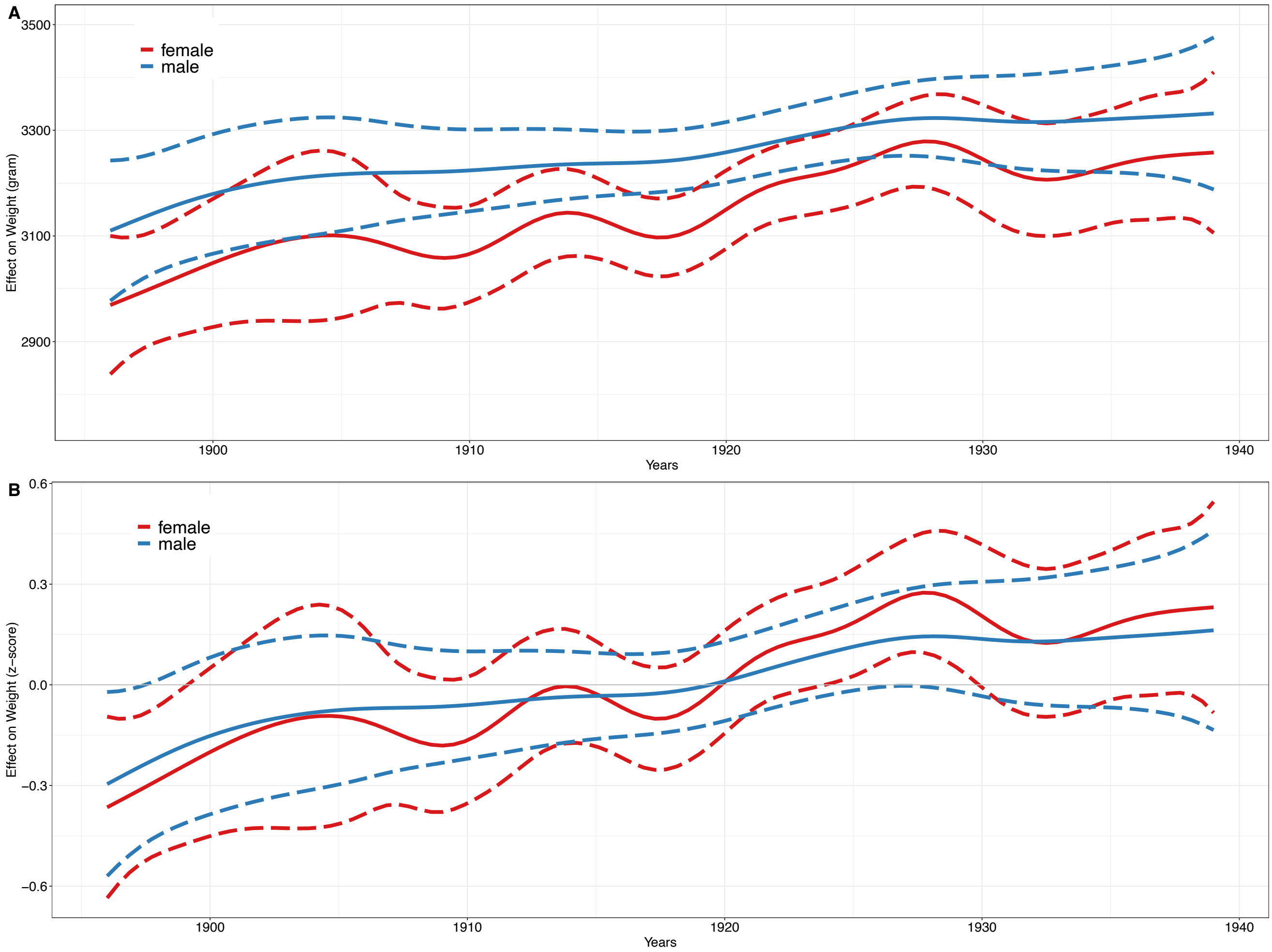

Figure S9: The adjusted model for the maternal height effect, stratified by sex. A) The dependent variable birthweight displayed as grams; B) the dependent variable birthweight displayed as z-scores.

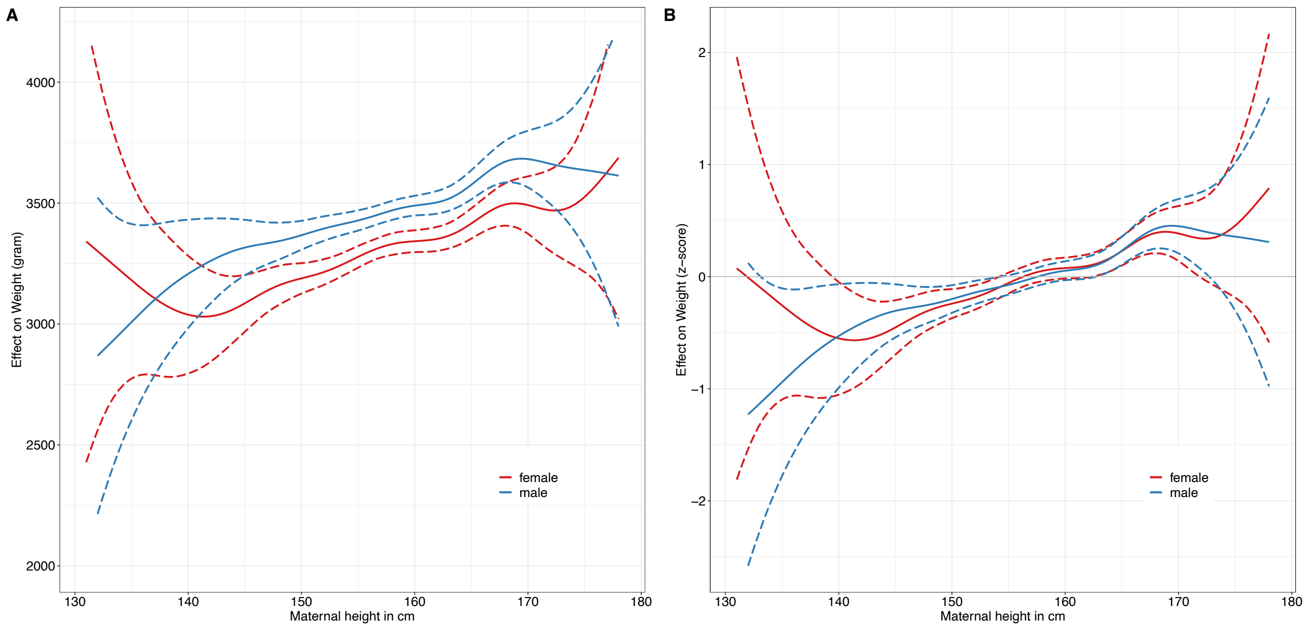

Table S1: Descriptive statistics for explanatory variables for all included years.

	1896 (N=523)	1897 (N=409)	1898 (N=397)	1899 (N=650)	1906 (N=195)	1907 (N=178)	1908 (N=179)	1909 (N=180)	1910 (N=193)	1911 (N=188)	1912 (N=135)	1913 (N=160)	1914 (N=226)	1915 (N=252)	1916 (N=297)	1917 (N=227)	1918 (N=246)	1919 (N=261)	1920 (N=198)
maternalheight																			
- Mean (SD)	155.1 (5.9)	154.9 (5.9)	154.9 (6.0)	155.2 (5.6)	156.0 (6.4)	156.3 (6.1)	156.7 (5.6)	156.6 (5.8)	156.4 (6.9)	156.5 (5.8)	156.1 (6.7)	157.5 (6.7)	157.0 (5.7)	156.7 (5.5)	158.2 (6.5)	158.3 (6.1)	158.2 (6.2)	158.4 (5.7)	158.0 (5.8)
- Range	133.0 - 174.0	138.0 - 175.0	136.0 - 171.0	135.0 - 174.0	134.0 - 173.0	140.0 - 173.0	134.0 - 175.0	142.0 - 175.0	132.0 - 172.0	131.0 - 176.0	132.0 - 171.0	135.0 - 175.0	136.0 - 178.0	140.0 - 170.0	134.0 - 173.0	141.0 - 174.0	145.0 - 175.0	142.0 - 171.0	141.0 - 172.0
birthweight																			
- Mean (SD)	3184.2 (483.7)	3206.3 (475.3)	3178.5 (462.2)	3224.7 (495.7)	3326.3 (477.6)	3240.9 (474.2)	3248.0 (504.1)	3225.4 (500.4)	3200.1 (516.4)	3260.0 (476.8)	3300.2 (492.4)	3339.2 (531.7)	3289.7 (479.0)	3317.3 (460.7)	3290.5 (478.3)	3259.4 (477.5)	3227.7 (483.7)	3235.9 (471.0)	3349.0 (446.4)
- Range	1250.0 - 5120.0	1800.0 - 4450.0	1520.0 - 4760.0	1400.0 - 4700.0	2050.0 - 4720.0	1840.0 - 5050.0	1980.0 - 4880.0	1770.0 - 5240.0	1900.0 - 4770.0	1720.0 - 4850.0	1950.0 - 4550.0	1290.0 - 4500.0	1530.0 - 4730.0	1670.0 - 4770.0	1820.0 - 4850.0	1450.0 - 4500.0	1520.0 - 5080.0	1480.0 - 4720.0	2150.0 - 4500.0
gestationalage																			
- Mean (SD)	39.9 (2.1)	39.8 (2.2)	39.6 (2.1)	39.7 (2.1)	39.7 (2.1)	39.9 (1.8)	39.8 (1.9)	39.8 (2.2)	39.7 (2.3)	39.9 (1.7)	39.9 (1.8)	40.1 (2.1)	40.0 (1.8)	40.1 (1.9)	39.7 (2.0)	39.8 (1.9)	40.0 (2.0)	40.1 (2.4)	40.2 (1.8)
- Range	32.0 - 44.9	29.7 - 44.7	31.7 - 44.7	27.9 - 44.9	29.3 - 44.6	33.7 - 43.6	30.0 - 43.9	23.4 - 43.7	26.0 - 44.7	33.4 - 44.1	33.1 - 44.3	30.0 - 44.1	33.3 - 44.9	32.1 - 44.6	32.0 - 44.6	30.7 - 44.4	30.3 - 44.9	29.1 - 44.7	33.3 - 44.4
sex																			
- 1	268 (51.2%)	213 (52.1%)	207 (52.1%)	329 (50.6%)	112 (57.4%)	89 (50.0%)	91 (50.8%)	92 (51.1%)	99 (51.3%)	88 (46.8%)	63 (46.7%)	87 (54.4%)	116 (51.3%)	127 (50.4%)	164 (55.2%)	115 (50.7%)	124 (50.4%)	144 (55.2%)	105 (53.0%)
- 2	255 (48.8%)	196 (47.9%)	190 (47.9%)	321 (49.4%)	83 (42.6%)	89 (50.0%)	88 (49.2%)	88 (48.9%)	94 (48.7%)	100 (53.2%)	72 (53.3%)	73 (45.6%)	110 (48.7%)	125 (49.6%)	133 (44.8%)	112 (49.3%)	122 (49.6%)	117 (44.8%)	93 (47.0%)
sep_comb_3a																			
- medium	307 (58.7%)	241 (58.9%)	229 (57.7%)	413 (63.5%)	114 (58.5%)	101 (56.7%)	101 (56.4%)	116 (64.4%)	106 (54.9%)	88 (46.8%)	61 (45.2%)	94 (58.8%)	136 (60.2%)	142 (56.3%)	179 (60.3%)	127 (55.9%)	135 (54.9%)	124 (47.5%)	113 (57.1%)
- low	181 (34.6%)	129 (31.5%)	147 (37.0%)	192 (29.5%)	60 (30.8%)	63 (35.4%)	58 (32.4%)	41 (22.8%)	54 (28.0%)	53 (28.2%)	49 (36.3%)	42 (26.2%)	59 (26.1%)	70 (27.8%)	63 (21.2%)	56 (24.7%)	62 (25.2%)	75 (28.7%)	57 (28.8%)
- high	28 (5.4%)	23 (5.6%)	16 (4.0%)	38 (5.8%)	21 (10.8%)	13 (7.3%)	20 (11.2%)	23 (12.8%)	31 (16.1%)	45 (23.9%)	23 (17.0%)	21 (13.1%)	28 (12.4%)	40 (15.9%)	50 (16.8%)	41 (18.1%)	47 (19.1%)	40 (15.3%)	26 (13.1%)
- housewife	2 (0.4%)	4 (1.0%)	0 (0.0%)	6 (0.9%)	0 (0.0%)	0 (0.0%)	0 (0.0%)	0 (0.0%)	0 (0.0%)	0 (0.0%)	0 (0.0%)	1 (0.6%)	3 (1.3%)	0 (0.0%)	4 (1.3%)	3 (1.3%)	1 (0.4%)	17 (6.5%)	1 (0.5%)
- missing	5 (1.0%)	12 (2.9%)	5 (1.3%)	1 (0.2%)	0 (0.0%)	1 (0.6%)	0 (0.0%)	0 (0.0%)	2 (1.0%)	2 (1.1%)	2 (1.5%)	2 (1.2%)	0 (0.0%)	0 (0.0%)	1 (0.3%)	0 (0.0%)	1 (0.4%)	5 (1.9%)	1 (0.5%)
Paritycat																			
- 1	195 (37.3%)	148 (36.2%)	185 (46.6%)	269 (41.4%)	66 (33.8%)	81 (45.5%)	78 (43.6%)	77 (42.8%)	88 (45.6%)	88 (46.8%)	51 (37.8%)	64 (40.0%)	114 (50.4%)	103 (40.9%)	121 (40.7%)	106 (46.7%)	106 (43.1%)	151 (57.9%)	105 (53.0%)
- 2	156 (29.8%)	102 (24.9%)	99 (24.9%)	174 (26.8%)	43 (22.1%)	33 (18.5%)	48 (26.8%)	49 (27.2%)	40 (20.7%)	51 (27.1%)	43 (31.9%)	41 (25.6%)	49 (21.7%)	65 (25.8%)	82 (27.6%)	58 (25.6%)	62 (25.2%)	49 (18.8%)	49 (24.7%)
- >=3	172 (32.9%)	159 (38.9%)	113 (28.5%)	207 (31.8%)	86 (44.1%)	64 (36.0%)	53 (29.6%)	54 (30.0%)	65 (33.7%)	49 (26.1%)	41 (30.4%)	55 (34.4%)	63 (27.9%)	84 (33.3%)	94 (31.6%)	63 (27.8%)	78 (31.7%)	61 (23.4%)	44 (22.2%)

	1921 (N=317)	1922 (N=261)	1923 (N=281)	1924 (N=283)	1925 (N=288)	1926 (N=230)	1927 (N=324)	1928 (N=303)	1929 (N=158)	1930 (N=174)	1931 (N=174)	1932 (N=198)	1933 (N=155)	1934 (N=312)	1935 (N=296)	1936 (N=273)	1937 (N=273)	1938 (N=154)	1939 (N=284)
maternalheight																			
- Mean (SD)	158.5 (5.6)	157.9 (6.3)	158.1 (6.3)	158.3 (5.4)	158.3 (5.9)	158.2 (5.7)	158.3 (5.9)	158.5 (6.2)	157.9 (5.7)	158.7 (5.7)	157.5 (5.3)	159.2 (6.0)	158.7 (6.0)	158.5 (5.8)	159.8 (5.7)	159.1 (5.7)	158.7 (6.0)	158.8 (5.6)	159.5 (5.8)
- Range	142.0 - 174.0	141.0 - 174.0	134.0 - 175.0	141.0 - 173.0	135.0 - 175.0	143.0 - 172.0	143.0 - 177.0	137.0 - 172.0	142.0 - 173.0	146.0 - 175.0	140.0 - 169.0	144.0 - 178.0	140.0 - 175.0	142.0 - 174.0	143.0 - 173.0	140.0 - 175.0	136.0 - 172.0	145.0 - 174.0	141.0 - 177.0
birthweight																			
- Mean (SD)	3302.0 (476.8)	3316.5 (485.1)	3321.6 (468.1)	3332.0 (453.1)	3314.7 (510.9)	3330.7 (489.5)	3394.8 (452.3)	3404.7 (472.5)	3333.0 (434.1)	3303.9 (452.2)	3255.1 (453.7)	3279.3 (477.0)	3345.8 (516.5)	3317.2 (505.4)	3324.6 (498.7)	3334.3 (501.2)	3335.7 (498.3)	3377.7 (416.3)	3334.5 (494.1)
- Range	1600.0 - 4690.0	2050.0 - 4700.0	1820.0 - 5080.0	1900.0 - 4500.0	1750.0 - 4820.0	1370.0 - 4500.0	2070.0 - 4950.0	2000.0 - 4950.0	2090.0 - 4700.0	1880.0 - 4480.0	1800.0 - 4330.0	1280.0 - 4500.0	1420.0 - 4600.0	1650.0 - 4700.0	1500.0 - 4820.0	1670.0 - 4800.0	1470.0 - 5890.0	2400.0 - 4580.0	1750.0 - 4800.0
gestationalage																			
- Mean (SD)	39.8 (1.8)	39.8 (2.1)	40.0 (1.8)	39.9 (1.8)	40.0 (1.9)	39.8 (1.7)	40.0 (1.9)	39.8 (1.8)	40.0 (1.8)	40.1 (1.9)	39.9 (2.0)	39.9 (2.0)	39.9 (2.0)	39.9 (2.1)	40.0 (2.0)	39.9 (1.9)	40.1 (1.9)	39.8 (1.8)	40.0 (1.6)
- Range	32.0 - 44.3	23.9 - 44.3	32.0 - 44.6	33.0 - 44.4	32.3 - 44.6	30.9 - 44.6	24.1 - 44.0	32.4 - 44.7	34.1 - 44.9	31.1 - 44.9	29.9 - 44.7	28.4 - 44.1	30.9 - 44.3	32.4 - 44.6	30.4 - 44.9	31.7 - 44.6	33.3 - 44.9	29.6 - 44.7	31.7 - 43.7
sex																			
- 1	159 (50.2%)	134 (51.3%)	134 (47.7%)	150 (53.0%)	142 (49.3%)	119 (51.7%)	173 (53.4%)	161 (53.1%)	84 (53.2%)	99 (56.9%)	91 (52.3%)	103 (52.0%)	85 (54.8%)	173 (55.4%)	154 (52.0%)	157 (57.5%)	129 (47.3%)	78 (50.6%)	144 (50.7%)
- 2	158 (49.8%)	127 (48.7%)	147 (52.3%)	133 (47.0%)	146 (50.7%)	111 (48.3%)	151 (46.6%)	142 (46.9%)	74 (46.8%)	75 (43.1%)	83 (47.7%)	95 (48.0%)	70 (45.2%)	139 (44.6%)	142 (48.0%)	116 (42.5%)	144 (52.7%)	76 (49.4%)	140 (49.3%)
sep_comb_3a																			
- medium	191 (60.3%)	141 (54.0%)	159 (56.6%)	159 (56.2%)	173 (60.1%)	127 (55.2%)	1 (0.3%)	2 (0.7%)	0 (0.0%)	0 (0.0%)	0 (0.0%)	133 (67.2%)	96 (61.9%)	197 (63.1%)	13 (4.4%)	19 (7.0%)	12 (4.4%)	85 (55.2%)	148 (52.1%)
- low	74 (23.3%)	84 (32.2%)	77 (27.4%)	77 (27.2%)	64 (22.2%)	61 (26.5%)	3 (0.9%)	0 (0.0%)	1 (0.6%)	0 (0.0%)	3 (1.7%)	48 (24.2%)	37 (23.9%)	77 (24.7%)	19 (6.4%)	15 (5.5%)	20 (7.3%)	47 (30.5%)	92 (32.4%)
- high	43 (13.6%)	34 (13.0%)	39 (13.9%)	32 (11.3%)	47 (16.3%)	34 (14.8%)	0 (0.0%)	0 (0.0%)	0 (0.0%)	0 (0.0%)	0 (0.0%)	16 (8.1%)	18 (11.6%)	32 (10.3%)	1 (0.3%)	0 (0.0%)	0 (0.0%)	22 (14.3%)	43 (15.1%)
- housewife	6 (1.9%)	1 (0.4%)	3 (1.1%)	6 (2.1%)	1 (0.3%)	0 (0.0%)	45 (13.9%)	69 (22.8%)	6 (3.8%)	0 (0.0%)	12 (6.9%)	0 (0.0%)	4 (2.6%)	6 (1.9%)	261 (88.2%)	238 (87.2%)	237 (86.8%)	0 (0.0%)	1 (0.4%)
- missing	3 (0.9%)	1 (0.4%)	3 (1.1%)	9 (3.2%)	3 (1.0%)	8 (3.5%)	275 (84.9%)	232 (76.6%)	151 (95.6%)	174 (100.0%)	159 (91.4%)	1 (0.5%)	0 (0.0%)	0 (0.0%)	2 (0.7%)	1 (0.4%)	4 (1.5%)	0 (0.0%)	0 (0.0%)
Paritycat																			
- 1	162 (51.1%)	136 (52.1%)	143 (50.9%)	142 (50.2%)	155 (53.8%)	106 (46.1%)	169 (52.2%)	174 (57.4%)	91 (57.6%)	104 (59.8%)	92 (52.9%)	106 (53.5%)	90 (58.1%)	165 (52.9%)	147 (49.7%)	138 (50.5%)	147 (53.8%)	78 (50.6%)	136 (47.9%)
- 2	77 (24.3%)	70 (26.8%)	72 (25.6%)	72 (25.4%)	85 (29.5%)	82 (35.7%)	82 (25.3%)	74 (24.4%)	42 (26.6%)	34 (19.5%)	46 (26.4%)	52 (26.3%)	37 (23.9%)	95 (30.4%)	83 (28.0%)	87 (31.9%)	74 (27.1%)	53 (34.4%)	93 (32.7%)
- >=3	78 (24.6%)	55 (21.1%)	66 (23.5%)	69 (24.4%)	48 (16.7%)	42 (18.3%)	73 (22.5%)	55 (18.2%)	25 (15.8%)	36 (20.7%)	36 (20.7%)	40 (20.2%)	28 (18.1%)	52 (16.7%)	66 (22.3%)	48 (17.6%)	52 (19.0%)	23 (14.9%)	55 (19.4%)

Table S2: Rating of explanatory factors from the birthweight GAM model (model 2)—this time with SEP as sensitivity analysis—sorted according to contribution to ΔAIC (Akaike's Information Criterion). The larger ΔAIC is, the more important the variable is in the model. Maternal height was the second most important explanatory variable after gestational age (as in the model without SEP).

**Parameter**	**Δ AIC**
Gestational age	1657.20
Maternal height	323.85
Parity	261.02
Sex	243.38
Birth year	42.21
Maternal year of birth	14.46
SEP	-0.21
